# Short Chain Fatty Acid Production from Mycoprotein and Mycoprotein Fibre in an In Vitro Fermentation Model

**DOI:** 10.3390/nu11040800

**Published:** 2019-04-08

**Authors:** Hannah C. Harris, Christine A. Edwards, Douglas J. Morrison

**Affiliations:** 1 School of Medicine, Dentistry and Nursing, College of Medical Veterinary and Life Sciences, University of Glasgow, Glasgow G31 2ER, UK; hannahcharris1@gmail.com (H.C.H.); Christine.Edwards@glasgow.ac.uk (C.A.E.); 2 Scottish Universities Environmental Research Centre, University of Glasgow, Glasgow G75 0QF, UK

**Keywords:** mycoprotein, Quorn, SCFA, colonic fermentation, dietary fibre

## Abstract

Dietary mycoprotein (marketed as Quorn^TM^) has many health benefits, including reductions in energy intake. The majority of studies evaluating mycoprotein focus on the protein content and very few consider the fibre content. Fibre consumption is also associated with decreased energy intake, which is partly attributed to short chain fatty acids (SCFAs) from fibre fermentation by colonic bacteria. To study the SCFA-producing capability of mycoprotein, in vitro batch fermentations were conducted, and SCFA production compared with that from extracted mycoprotein fibre, oligofructose (OF), rhamnose, and laminarin. Mycoprotein and mycoprotein fibre were both fermentable, resulting in a total SCFA production of 24.9 (1.7) and 61.2 (15.7) mmol/L, respectively. OF led to a significantly higher proportion of acetate compared to all other substrates tested (92.6 (2.8)%, *p* < 0.01). Rhamnose generated the highest proportion of propionate (45.3 (2.0)%, *p* < 0.01), although mycoprotein and mycoprotein fibre yielded a higher proportion of propionate compared with OF and laminarin. Butyrate proportion was the highest with laminarin (28.0 (10.0)% although mycoprotein fibre led to a significantly higher proportion than OF (*p* < 0.01). Mycoprotein is a valuable source of dietary protein, but its fibre content is also of interest. Further evaluation of the potential roles of the fibre content of mycoprotein is required.

## 1. Introduction

Mycoprotein, marketed as Quorn^TM^, is an animal meat protein substitute product formed from the cultivation of the microfungus *Fusarium venenatum*. Mycoprotein is high in protein (11% wet weight), low in fat (3% wet weight), and contains 6% dietary fibre by wet weight [1]. Mycoprotein consumption has been associated with improvements in plasma cholesterol, glucose and insulin, and energy intake, compared with other protein sources [2,3,4].

Dietary fibre has also been associated with reduced energy intake [5] and improved glucose metabolism. Fibre is fermented by colonic microbiota, resulting in the production of short chain fatty acids (SCFAs), namely, acetate, propionate, and butyrate. These SCFAs activate the free fatty acid receptors (FFAR) 2 and 3, which are present on a number of different cell types, such as the colonic L-cells, and have been associated with improved metabolic and immune regulation [6]. When delivered to the colon, propionate, in particular, has been associated with improved appetite regulation and weight gain control which occurs, at least in part, through a FFAR2-mediated mechanism involving the release of the anorectic gut hormones GLP-1 and PYY [7].

Two-thirds of the dietary fibre content within mycoprotein is β-glucan consisting of β(1–3), and β(1–6) bonding, with the remaining third in the form of chitin (*N*-acetyl glucosamine monomers bound by β(1–4) linkages) [1]. These bonding linkages occur in a variety of food sources, such as seaweed (containing laminarin) [8], mushrooms [9], and oats [10], all of which have been shown to be fermentable and produce SCFA in vitro. The SCFA-producing capability of mycoprotein is currently unknown. Understanding SCFA production from mycoprotein may help in elucidating the mechanisms by which mycoprotein delivers health benefits.

## 2. Materials and Methods

### 2.1. Substrates

Mycoprotein (whole) in the form of Chicken Style Pieces, and isolated mycoprotein fibre powder from an in-house purification method (>75% fibre) was provided by Marlow Foods (Stokesley, UK). Briefly, mycoprotein was digested with 20× (*vol/wt*) of 0.5 M NaOH and residual carbohydrate precipitate was washed and freeze-dried to produce a fibre-rich extract. Rhamnose, laminarin (both from Sigma, Poole, UK), and oligofructose (OF) (Beneo P95) were used for comparison.

### 2.2. Batch Fermentation

The in vitro fermentations were carried out as previously described [11] in 10 mL fermentation vessels containing 100 mg of substrate, a medium containing 200 µL reducing solution, and 4.2 mL of a pre-boiled, pre-reduced fermentation medium consisting of micronutrients, macronutrients, and 0.1% resazurin. The medium was adjusted to pH 7, the vessels sealed to be airtight, and the contents further reduced with oxygen-free nitrogen for one minute. A 32% faecal slurry was prepared by adding pre-boiled, oxygen-free nitrogen cooled sodium phosphate Sorensen’s buffer to homogenised stool and then blended and strained to remove particulates prior to injection (500 µL) into the vessel. The vessels were further reduced until they were anaerobic (as indicated by resazurin) using oxygen-free nitrogen, and incubated in a shaking water bath at 37 °C. After 0 and 24 h of incubation, an 800 μL aliquot of fermentation fluid was taken for pH measurement (Mettler Toledo pH meter), and 300 μL of 1 M NaOH added to stabilise the SCFA before extraction and analysis as described in [12]. Data are presented as means (±SEM) and statistical analysis was performed using ANOVA with post hoc Tukey HSD, as the data were deemed to be normally distributed based on the Shapiro–Wilk test using SPSS version 22 (IBM, Armonk, NY, USA).

### 2.3. Participants

Stool samples were obtained from three healthy Caucasian individuals (1 female, 2 males, aged 24–25 years), who had not taken antibiotics during the previous 6 months. Ethical permission was granted by the University of Glasgow, College of MVLS, Ethics Committee (ref: 2011023). Faecal donors provided signed informed consent.

## 3. Results

### 3.1. SCFA Production (Table 1)

All of the tested substrates were fermentable, with whole mycoprotein resulting in the lowest total SCFA production (24.9 (1.7) mmol/L). The fibre fraction of the mycoprotein resulted in the second highest total SCFA production at 61.2 (15.7) mmol/L, being surpassed only by laminarin (64.1 (9.0) mmol/L). The concentrations of acetate, propionate, and butyrate are shown in Table S1.

The highest proportion of acetate was produced with OF fermentation (92.6 (2.8)%, *p* < 0.01). By contrast, OF was associated with the lowest proportion of propionate (2.6 (0.3)%), whilst rhamnose led to the highest proportion of propionate production (45.3 (2.0)%, *p* < 0.01). Whole mycoprotein and mycoprotein fibre both resulted in a significantly higher proportion of propionate production compared to both OF and laminarin (26.0 (3.5)% and 20.3 (1.3)% vs. 2.6 (0.3)% and 7.0 (2.5)% respectively, *p* < 0.001 for both). Laminarin fermentation generated the greatest proportion of butyrate (28.0 (10.0)%), although whole mycoprotein also led to increased butyrate when compared to OF (*p* < 0.01).

### 3.2. Ranking of Substrates (Table 2)

Variability between the SCFAs from each stool donor was noted, for example, the amount of SCFA produced from mycoprotein fibre ranged from 37.5 to 90.9 mmol/L. SCFA production was ranked for each substrate and OF, laminarin, and mycoprotein fibre all ranked within the top three for acetate production (though the exact order differed for each stool donor). Rhamnose and mycoprotein fibre ranked first and second for propionate production in all three donors while laminarin was ranked first for butyrate production for all three donors and mycoprotein fibre ranked second for two of the three stool donors.

## 4. Discussion

Mycoprotein (commercially known as Quorn^TM^) is marketed as a sustainable high-quality alternative protein source to animal protein due to its high protein content (11% wet weight). Its protein content has been associated with many beneficial health effects such as reduced energy intake, and improved plasma insulin and cholesterol [2,3,4,13,14]. However, these health benefits may also result from the fibre content of mycoprotein.

Mycoprotein is composed of 6% fibre, comprised of chitin and β-glucan. β-Glucan is fermentable, leading to the production of SCFA, and has been shown to reduce plasma cholesterol [15]. Chitin is not commonly consumed in Western diets, and studies on the effect of chitins on the human gastrointestinal tract and on the gut-mediated effects on innate and adaptive immunity are lacking. However, chitosan, the soluble fraction of chitin, has been shown to reduce energy intake and total cholesterol in overweight individuals consuming 3 g/day and hypercholesterolemic individuals consuming 1.2 g/day [16,17]. A 75 g serving of mycoprotein contains 1.5 g of chitin and 4 g of β-glucan, indicating that both chitin and β-glucan may have an effect.

Here, we have demonstrated that whole mycoprotein pieces and the isolated fibre fraction are both fermentable in vitro, and lead to the production of SCFAs. Whole mycoprotein fermentation led to the production of 24.9 (1.7) mmol/L, which was the lowest of all of the substrates tested. By contrast, mycoprotein fibre led to the production of 61.2 (15.7) mmol/L which was significantly higher than that of the control 11.1 (0.9) mmol/L, and did not significantly differ from any of the other fermentable substrates tested. Within this study the total amount of SCFA produced was slightly different than previously noted; for example, Gietl et al. observed that rhamnose led to the production of 60.7 mmol/L total SCFA compared with 51.1 mmol/L in this study [18].

Whole mycoprotein contains 6% dietary fibre, and the mycoprotein fibre was 75% dietary fibre, indicating that whole mycoprotein led to 5 times more SCFA than mycoprotein fibre (per gram). No predigestion step was performed on any of the substrates tested prior to in vitro fermentation, and it is unlikely that the whole mycoprotein would be completely indigestible within the small intestine, indicating that products other than fibre may have undergone fermentation, resulting in the production of SCFA.

When the proportions of acetate, propionate, and butyrate were considered, whole mycoprotein led to significantly higher proportions of propionate compared with inulin and laminarin, but significantly lower than rhamnose, and with significantly higher levels of butyrate compared to rhamnose and oligofructose. Mycoprotein fibre led to significantly higher proportions of propionate compared with inulin and laminarin, but lower than rhamnose. Whole mycoprotein led to the second highest proportion of propionate (26.0 (3.5)%), second only to rhamnose (45.3 (2.0)%), which is considered propiogenic in vitro [18]. Mycoprotein also ranked second for butyrate production, second only to laminarin. This preference for propionate and butyrate production is likely due to the β-glucan content, which has been previously shown to result in higher in vitro propionate and butyrate production [10]. This preference is also of potential importance, as both are able to stimulate the release of PYY and GLP-1 by activating the FFAR2 and FFAR3 receptors found within colonic enteroendocrine cells [6]. Targeted delivery of ~2.5 g/day of propionate in the form of an inulin propionate ester (10 g/day) to overweight individuals and 5% (*w*/*w*) butyrate to mice both inhibit weight gain and reduce energy intake [7,19]. Whether this selective increased production of propionate from mycoprotein and mycoprotein fibre explains some of the health benefits of mycoprotein consumption remains to be elucidated.

## 5. Conclusions

We have shown that mycoprotein, and the purified dietary fibre fraction of mycoprotein are fermentable and produce SCFA. Mycoprotein and mycoprotein fibre appeared to promote propionate and butyrate production at the expense of acetate. The role of mycoprotein fibre in the observed health benefits from mycoprotein warrants further investigation.

## Figures and Tables

**Table 1:** Production of short chain fatty acid (SCFA) after 24 h batch fermentation.

		Ratio (% of SCFA Produced)		
Substrate	Total SCFA Production (mmol/L)	Acetate	Propionate	Butyrate
Control (blank)	11.13 (0.93) ^bcdf^	56.91 (6.35) ^b^	20.12 (2.17) ^bcd^	22.97 (3.88) ^b^
Oligofructose	50.05 (4.35) ^a^	92.57 (2.82) ^acdef^	2.57 (0.34) ^acef^	4.86 (2.72) ^ade^
Rhamnose	51.14 (1.90) ^a^	44.77 (3.13) ^b^	45.33 (1.95) ^abdef^	9.90 (2.70) ^de^
Laminarin	64.08 (9.0) ^a^	65.04 (11.51) ^b^	6.95 (2.48) ^acef^	28.02 (10.04) ^bc^
Mycoprotein (whole)	24.87 (1.68) ^d^	48.31 (6.53) ^b^	25.99 (3.51) ^bcd^	25.70 (3.11) ^bc^
Mycoprotein fibre	61.15 (15.73) ^a^	61.36 (4.17) ^b^	20.25 (1.28) ^bcd^	18.39 (5.18) ^g^

Mean (SEM), *n* = 3. Total SCFA production was calculated as the sum of acetate, propionate, and butyrate. Ratio calculated as a percentage of the total production. Superscript letters indicate significant difference from corresponding substrate where ^a^—control, ^b^—oligofructose, ^c^—rhamnose, ^d^—laminarin, ^e^—mycoprotein (whole), ^f^—mycoprotein fibre, ^g^—not significantly different from any other substrate.

**Table 2:** Ranking of SCFA production in in vitro cultures for each stool donor.

	Acetate			Propionate			Butyrate		
Rank	P1	P2	P3	P1	P2	P3	P1	P2	P3
1	OF	Laminarin	Mycoprotein fibre	Rhamnose	Rhamnose	Rhamnose	Laminarin	Laminarin	Laminarin
2	Laminarin	OF	OF	Mycoprotein fibre	Mycoprotein fibre	Mycoprotein fibre	Mycoprotein fibre	Mycoprotein (whole)	Mycoprotein fibre
3	Mycoprotein fibre	Mycoprotein fibre	Laminarin	Mycoprotein (whole)	Mycoprotein (whole)	Laminarin	Mycoprotein (whole)	Rhamnose	Mycoprotein (whole)
4	Rhamnose	Rhamnose	Rhamnose	Laminarin	Laminarin	Mycoprotein (whole)	Rhamnose	Mycoprotein fibre	Rhamnose
5	Mycoprotein (whole)	Mycoprotein (whole)	Mycoprotein (whole)	Control	Control	Control	OF	OF	Control
6	Control	Control	Control	OF	OF	OF	Control	Control	OF

P1 = stool donor 1, P2 = stool donor 2, P3 = stool donor 3. Rank number 1 = top, Rank number 6 = bottom. OF—oligofructose.

## Supplementary Materials

The following are available online at https://www.mdpi.com/2072-6643/11/4/800/s1, Table S1: Production of SCFA after 24 h batch fermentation.
