# Supplementary material for: Short Chain Fatty Acid Production from Mycoprotein and Mycoprotein Fibre in an In Vitro Fermentation Model
Source: Nutrients. 2019 Apr 8;11(4):800. doi: 10.3390/nu11040800 (PMC6520856; doi:10.3390/nu11040800)
Supplement: Supplementary file 1 [file nutrients-11-00800-s001.pdf]

nutrients

Supplementary Table 1: Production of SCFA after 24 hours batch fermentation.

	mmol/l		
	Acetate	Propionate	Butyrate
Control	6.45 (1.18)[bdf]	2.19 (0.11)[cf]	2.49 (0.19)[d]
Oligofructose	46.47 (4.91)[ade]	1.28 (0.13)[cf]	2.30 (0.64)[d]
Rhamnose	22.91 (1.40)	23.21 (1.33)[abdef]	5.02 (0.69)[d]
Laminarin	41.02 (4.53)[ae]	4.71 (1.64)[cf]	18.36 (5.54)[abc]
Mycoprotein	12.10 (1.57)[bdf]	6.43 (0.48)[c]	6.34 (0.31)[g]
Mycoprotein fibre	37.62 (10.28)[ae]	12.25 (2.98)[abcd]	11.28 (3.0)[g]

Mean (SEM), n = 3. Letters indicate significant difference from corresponding substrate; a – control, b – oligofructose, c – rhamnose, d – laminarin, e – mycoprotein (whole), f – mycoprotein fibre, g – not significantly different from any other substrate
